# The Syndromic versus Laboratory Diagnosis of Sexually Transmitted Infections in Resource-Limited Settings

**DOI:** 10.1155/2014/103452

**Published:** 2014-03-05

**Authors:** Musie Ghebremichael

**Affiliations:** Harvard Medical School and Ragon Institute of MGH, MIT and Harvard, 400 Technology Square, Room 857, Cambridge, MA 02129, USA

## Abstract

Sexually transmitted infections (STIs) are highly prevalent in sub-Saharan Africa, where there is a severe HIV epidemic. Thus, accurate recognition and diagnosis of STIs are essential for successful HIV prevention programs in the region. Due to lack of trained personnel and adequate laboratory infrastructure in the region, information regarding the profile of STIs relies essentially on self-reported or physician-diagnosed symptoms. The main objective of the study was to assess the effectiveness of the syndromic diagnosis of STIs, which is often used as a proxy for laboratory diagnosis of STIs in sub-Saharan Africa and other resource-limited settings. The study builds on previously collected data from a community-based survey in Northern Tanzania. We found no significant agreements between patient-reported STIs symptoms and laboratory-confirmed STIs tests. The reported STIs symptoms had high specificity (range = 85–99%) and poor sensitivity (range = 2–17%). Knowledge gained from our study will have significant public health implications, and can help improve the syndromic diagnosis of STIs.

## 1. Introduction

AIDS is a major public health challenge in sub-Saharan Africa, and the prevalence of other STIs in the region is high [1, 2]. An estimated 22.9 million people infected with HIV live in sub-Saharan Africa, and approximately 1.2 million deaths from AIDS occurred in sub-Saharan Africa [3]. There is compelling evidence that STIs affect the transmission of HIV. STIs facilitate the sexual acquisition and transmission of HIV infection and HIV infection increases the risk of other STIs [4, 5]. Hence, the recognition, treatment, and prevention of STIs to reduce the risk of HIV transmission should be a public health priority, especially in sub-Saharan Africa where antiretroviral therapy may not be readily available. More than two-thirds of patients living with HIV in sub-Saharan Africa have no access to antiretroviral therapy [6].

STIs can be diagnosed in a number of different ways, including laboratory and syndromic diagnosis. Laboratory diagnosis is the most accurate method of making a diagnosis. However, it is expensive and is not feasible in many settings as it requires sophisticated laboratory facilities and qualified personnel who can perform technically demanding procedures. Moreover, laboratory diagnosis of STIs is time-consuming and results often cannot be made available at the same visit, thereby causing a delay in treatment initiation. For these reasons, the syndromic diagnosis of STIs remains the only feasible option in some resource-limited settings. Syndromic diagnosis of STIs is based on the identification of a group of symptoms and signs that characterize a clinical condition [7, 8]. It is simple, cost effective, and capable of yielding rapid diagnosis for immediate treatment. Moreover, this technique can be implemented at all levels of the health care system. Despite these advantages, it has several limitations: syndromic diagnosis relies on subjective judgment, cannot detect asymptomatic infections, and may result in overdiagnosis/overtreatment and potential drug resistance [9–11].

Due to a lack of trained personnel and adequate laboratory infrastructure in sub-Saharan Africa, information regarding the profile of STIs relies essentially on self-reported or physician-diagnosed STIs. However, there is still no consensus on its performance and several studies from the region have reported inconsistent results. The mixed reports might be due to the fact that the studies were carried out in particular groups that are not representative of the overall population, such as STD or antenatal clinic attendees [12–14]. Therefore, there is a public health need to evaluate the validity of the syndromic diagnosis of STIs using population-based studies from the region, that is, the urgent need for a comparative study of syndromic and laboratory diagnosis of STIs in sub-Saharan Africa. The main objective of the study was to evaluate the performance of syndromic diagnosis of STIs in comparison with laboratory-confirmed STIs.

## 2. Materials and Methods

### 2.1. Study Design and Study Sample

The study participants consisted of 2,019 women who were enrolled in a community-based survey, which was conducted from November 2002 to March 2003 in the Moshi Urban District of the Kilimanjaro Region. The Kilimanjaro Region, located in Northern Tanzania adjacent to the Kenyan border, is one of Mainland Tanzania's 20 regions and is experiencing a mature generalized stage of the HIV epidemic. Tanzania, with a population of 34.5 million, has about 7% of its adults infected with HIV [15]. In the Moshi District of Northern Tanzania, the prevalence of HIV infection is about twice that of the national average.

Study participants were selected to participate in the survey based on a two-stage sampling design. During the first stage of sampling, a total of 150 clusters were selected from the Moshi Urban District. In the second stage of sampling, a number of households were randomly selected from each of the 150 clusters and 2,019 women who were residents of the households were enrolled. A detailed description of the study protocol, data gathering instruments, and procedures and laboratory methods has been previously published [16, 17].

### 2.2. Study Variables

Demographic and socioeconomic characteristics including age, education, ethnicity, religion, and occupation were obtained. High-risk behaviors including alcohol abuse, age at first sex, number of sexual partners, and frequency of condom use were obtained. Symptoms of STIs including abdominal pain, abnormal genital discharge, foul smell in the genital area, excessive genital secretions, swelling of lymphnodes in the genital area, itching in the genital area, burning pain on micturation, pain during intercourse, and genital ulcers were obtained. Blood and urine samples were obtained from 1,418 and 1,440 women, respectively, who agreed to further testing for STIs. The blood samples were tested for HIV-1, HSV-2, and active and past syphilis. The urine samples were tested for chlamydia, gonorrhea, *Trichomonas*, and *Mycoplasma genitalium.*

### 2.3. Statistical Analysis

Descriptive measures (such as mean, median, standard deviation, interquartile range, frequencies, and percentages) were used to summarize the data. Sensitivity and specificity together with their corresponding 95% confidence intervals were calculated to assess the predictive accuracy of each STIs symptom. Exact binomial confidence intervals were used to estimate confidence intervals for rates of STIs, sensitivities, and specificities. Kendall's tau-*b* was used to measure agreements between patient-reported STIs symptoms and laboratory confirmed STIs. The reported numbers of STIs symptoms were summed to obtain the total number of STIs symptoms for each participant. Receiver operating characteristic (ROC) curve was then constructed using sensitivity and specificity obtained from every possible symptom threshold. The area under the ROC curve (AUROC) was used to evaluate the overall diagnostic accuracy of the syndromic diagnosis of STIs. Bootstrap-based confidence intervals were calculated for the AUROC.

## 3. Results

We restricted our analysis to women who were tested for STIs; a total of 1,520 women were tested for STIs. The median age was 28 years (IQR = 23–35), and median age at first sex was 19 years (IQR = 17–21). The majority of study participants had pre-secondary education (77%), had not used a condom in the prior 12 months (77%), had one sex partner in the last three years (84%), and had a husband/partner (61%). Fifty percent of the women tested positive for at least one STI.

**Figure 1:**
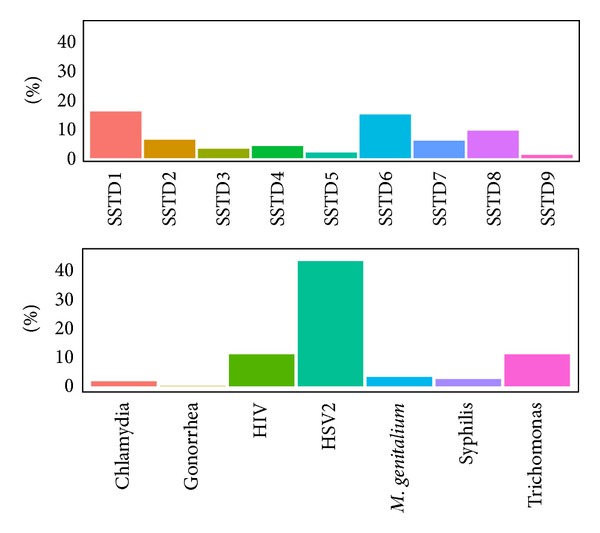
Prevalence of self-reported STIs symptoms and laboratory confirmed STIs (SSTD1: abdominal pain, SSTD2: abnormal genital discharge, SSTD3: foul smell in the genital area, SSTD4: excessive genital secretions, SSTD5: swelling of lymphnodes in the genital area, SSTD6: itching in the genital area, SSTD7: burning pain on micturation, SSTD8: pain during intercourse, and SSTD9: genital ulcers).

**Figure 2:**
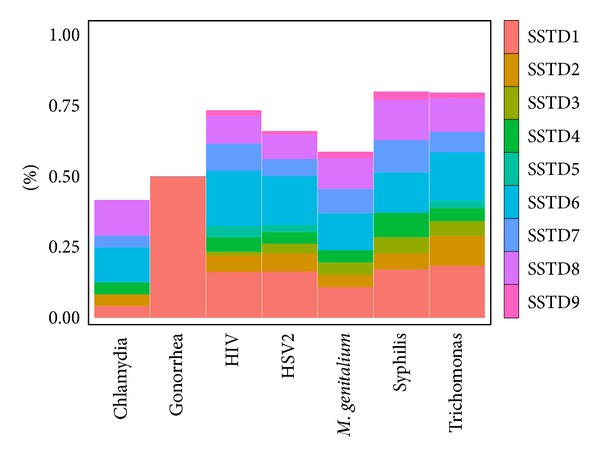
Prevalence of self-reported STIs symptoms among women tested positive for STIs (SSTD1: abdominal pain, SSTD2: abnormal genital discharge, SSTD3: foul smell in the genital area, SSTD4: excessive genital secretions, SSTD5: swelling of lymphnodes in the genital area, SSTD6: itching in the genital area, SSTD7: burning pain on micturation, SSTD8: pain during intercourse, and SSTD9: genital ulcers).

Figure 1 displays the rates of STIs symptoms and tests among the women included in the analysis. The prevalence rates of HSV-2, HIV-1, and *Trichomonas* were 43%, 11%, and 11%, respectively. The prevalence rates of the other STIs were below 5.0%. Among the tested women, 30% reported at least one STIs symptom. The most prevalent STIs symptom was lower abdominal pain (16%), followed by itching in the genital area (15%), pain during intercourse (9.4%), abnormal genital discharge (6.3%), burning or pain on micturation (6%), excessive genital secretions (4.1%), foul smell in genital area (3.2%), swelling of lymphnodes in genital area (1.9%), and genital ulcers (1.1%). The prevalence of STIs symptoms among women tested positive for each STI is presented in Figure 2. Multiple STIs symptoms were reported with almost all the laboratory-confirmed STIs. Lower abdominal pain was a common symptom of several infections.

**Figure 3:**
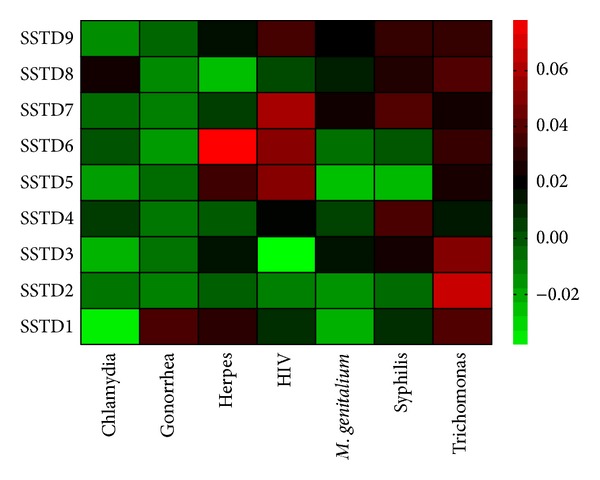
Heat map of Kendall's tau-*b* correlation coefficients between STIs symptoms and laboratory confirmed STIs (SSTD1: abdominal pain, SSTD2: abnormal genital discharge, SSTD3: foul smell in the genital area, SSTD4: excessive genital secretions, SSTD5: swelling of lymphnodes in the genital area, SSTD6: itching in the genital area, SSTD7: burning pain on micturation, SSTD8: pain during intercourse, and SSTD9: genital ulcers).

Figure 3 displays a heat map of Kendall's tau-*b* correlation coefficients used to measure agreements between patient-reported STIs symptoms and laboratory-confirmed results. Kendall's tau-*b* value of 1 implies perfect agreement and values less than 1 imply less than perfect agreement. The Kendall's tau-*b* coefficients were low (range = −0.04–0.08), showing no significant associations between each STI symptom and the laboratory-confirmed STI results. When the symptoms were combined together and summed, the level of agreement between STIs symptoms and tests remained low (Kendall's tau-*b* = 0.07, 95% CI: 0.02–0.12).

**Figure 4:**
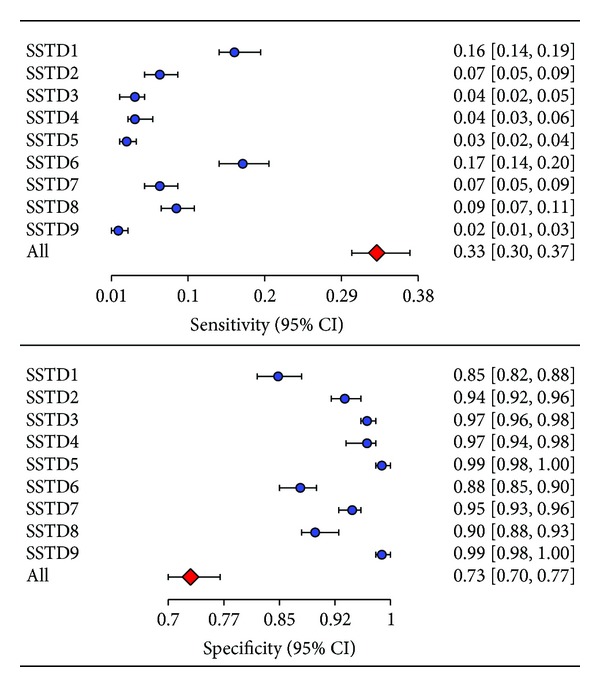
Sensitivities and specificities of STIs symptoms together with their 95% exact confidence intervals (SSTD1: abdominal pain, SSTD2: abnormal genital discharge, SSTD3: foul smell in the genital area, SSTD4: excessive genital secretions, SSTD5: swelling of lymphnodes in the genital area, SSTD6: itching in the genital area, SSTD7: burning pain on micturation, SSTD8: pain during intercourse, and SSTD9: genital ulcers).

**Figure 5:**
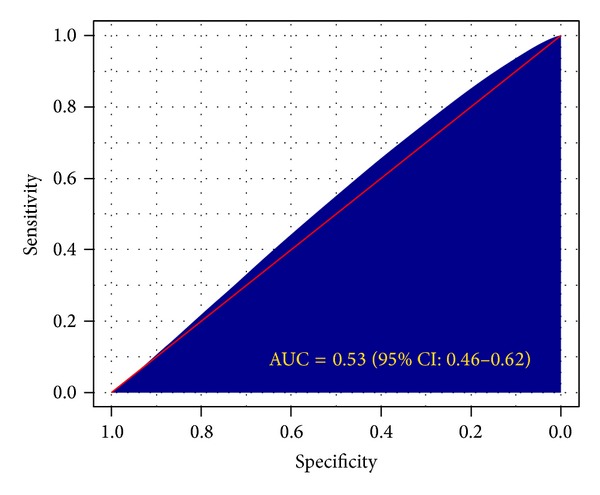
The receiver operating characteristics (ROC) curve. The shaded region represents the area under the ROC curve.

Figure 4 displays the sensitivity (true positive rate) and specificity (true negative rate) of each STI symptom, where sensitivity is the ability of an STI symptom to correctly identify women who have laboratory-confirmed STIs and specificity is the ability of an STI symptom to correctly identify women without laboratory-confirmed STIs. The individual STI symptoms had very low sensitivity (range = 2%–17%) and high specificity (range = 85%–99%). The less sensitive STI symptoms identified individuals as being disease-free when in fact they were not. Moreover, the more specific STIs symptoms identified individuals as being disease-free when they were disease-free. Figure 4 also displays the sensitivity and specificity of the combined STIs symptoms, which was defined as a positive response to at least one of the reported symptoms. The sensitivity and specificity of the combined STIs symptoms were 0.33 [95% CI: 0.30–0.37] and 0.73 [95% CI: 0.70–0.77]. The combined STIs symptoms correctly identified 33 out of 100 women with laboratory-confirmed STIs and resulted in a 67% false negative rate. Similarly, the combined STIs symptoms correctly identified 73 out of 100 healthy women and resulted in 27% false positive rate.  Figure 5 displays the area under the receiver-operating curve (AUROC). The AUROCmeasures the overall diagnostic accuracy of the STI symptoms. The closer the AUROC value is to 1, the better the syndromic diagnosis of STIs is. The AUROC was 0.53 (95% CI: 046–0.62); that is, the syndromic approach had a 53% probability of correctly distinguishing a healthy from an infected woman. The 95% CI included 50%, indicating that there is no gain in predictive accuracy by using STIs symptoms. The syndromic approach showed no advantage over random guess (a random classifier has an AUROC of 0.5).

## 4. Discussion

Our study aimed to evaluate the accuracy (performance) of syndromic management of sexually transmitted infections (STIs) among women in sub-Saharan Africa. It included 1,520 women who were enrolled in a community-based survey from the Moshi Urban District of Northern Tanzania. Half of the women tested positive for at least one STI, while 30% reported at least one STIs symptom. The most prevalent STI among the study participants was HSV-2 (reaching 43%), followed by HIV and *Trichomonas* infection rates of 11%. The prevalence rates of other STIs were *Mycoplasma Genitalium *(3.2%), syphilis (2.5%), chlamydia (1.8%), and gonorrhea (0.2%). The most prevalent STI symptom was lower abdominal pain (16%), followed by itching in the genital area (15%), pain during intercourse (9%), abnormal genital discharge (6%), and burning or pain on micturation (6%). The prevalence rates of the remaining STIs symptoms were below 5.0%. We found no significant associations between patient-reported STIs symptoms and laboratory-confirmed STIs tests. Furthermore, we found that the individual STIs symptoms had high specificity and poor sensitivity. The sensitivity (specificity) of each symptom independently ranged from 2 to 17% (85–99%). The less sensitive STIs symptoms identified women as being infection-free when in fact they were not (high false-negative results). The more specific STIs symptoms identified women as infection-free when they were infection-free (low false-positive results). When the individual STIs were used together, the combined STIs symptoms showed poor sensitivity of 33% and moderate specificity of 73%. Nearly 67% of the infected women were missed and almost 27% of the women who report STIs symptoms were infection-free. The choice of less sensitive versus more specific tests depends on whether one wants to exclude a dangerous disease or avoid a dangerous therapy. A test's sensitivity (specificity) becomes particularly important when one is seeking to exclude a dangerous disease (to confirm a diagnosis that requires dangerous therapy).

Several previous studies from sub-Saharan African countries and other parts of the world have reported inconsistent results on the performance of STIs symptoms. Clark et al. [18] evaluated the diagnostic performance of self-reported STIs symptoms among high-risk men and women in Peru. The authors reported that the STIs symptoms had low sensitivity and high specificity consistent with our findings. Low sensitivity and high specificity of self-reported STIs symptoms were also reported in a Chinese study by Yin et al. [19]. A study by Mukenge-Tshibaka et al. [13] among female sex workers in Benin reported poor sensitivity of the STIs symptoms. Studies in Egypt and India [20, 21] among women attending antenatal, family planning, and peripheral government clinics reported high sensitivity and low specificity of STIs symptoms. In a study conducted in South Africa among women attending STD clinic, the sensitivity of STIs symptoms varied greatly, ranging from 0% to 88%, in detecting different infections [14].

Our study has several strengths compared to previous studies. It is a large population-based study, enhancing its generalizability. Most studies on the association between STIs symptoms and tests have been carried out in particular groups that are not representative of the overall population, such as STD or antenatal clinic attendees, or sex workers [13, 14, 20–22]. However, this study also had some limitations that may have influenced our findings. First, only women who agreed to be tested for STIs were included in the analysis, thereby ignoring the possible systematic differences between women who consented to be tested for STIs and those who did not. Analysis of the complete cases can be biased if there was a systematic difference between cases with observed data and those with unobserved data. For example, if women without or with fewer STIs symptoms consented to be tested, this might have led to an underestimation of the true association between STIs symptoms and tests. Second, some of the reported symptoms could have been caused by infections not measured in the study. This might have also led to an underestimation of the true association between STIs symptoms and tests. Third, the accuracy of the information given may be affected by the sensitive nature of information regarding symptoms of STIs. Thus, the self-reported STIs symptoms might have introduced social desirability and recall biases and were likely to be underreported.

The syndromic diagnosis of STIs is often used in resource-poor settings where laboratory diagnosis is limited. It is simple, cost effective, and capable of yielding rapid diagnosis for immediate treatment. However, it should be validated using population-based data and periodically evaluated.
